# Supplementary material for: Seminoma and Embryonal Carcinoma Footprints Identified by Analysis of Integrated Genome-Wide Epigenetic and Expression Profiles of Germ Cell Cancer Cell Lines
Source: PLoS One. 2014 Jun 2;9(6):e98330. doi: 10.1371/journal.pone.0098330 (PMC4041891; doi:10.1371/journal.pone.0098330)
Supplement: File S1 — ZIP file containing DMRforPairs output for significant regions. Please start from the html files. (ZIP) [file pone.0098330.s008.zip › figures/4668.pdf]

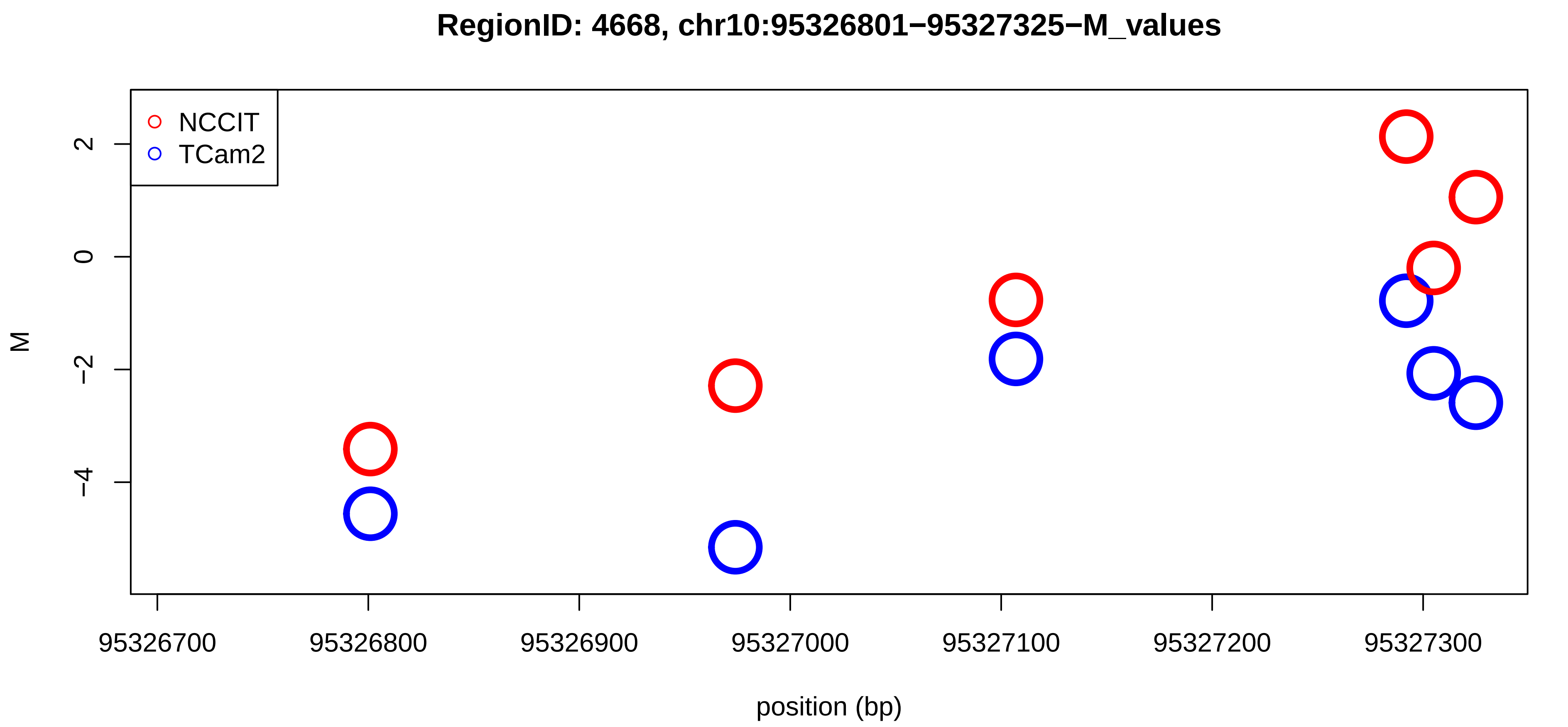
RegionID: 4668, chr10:95326801−95327325−M_values
NCCIT
TCam2
M
2
0
−2
−4
95326700
95326800
95326900
95327000
95327100
95327200
95327300
position (bp)

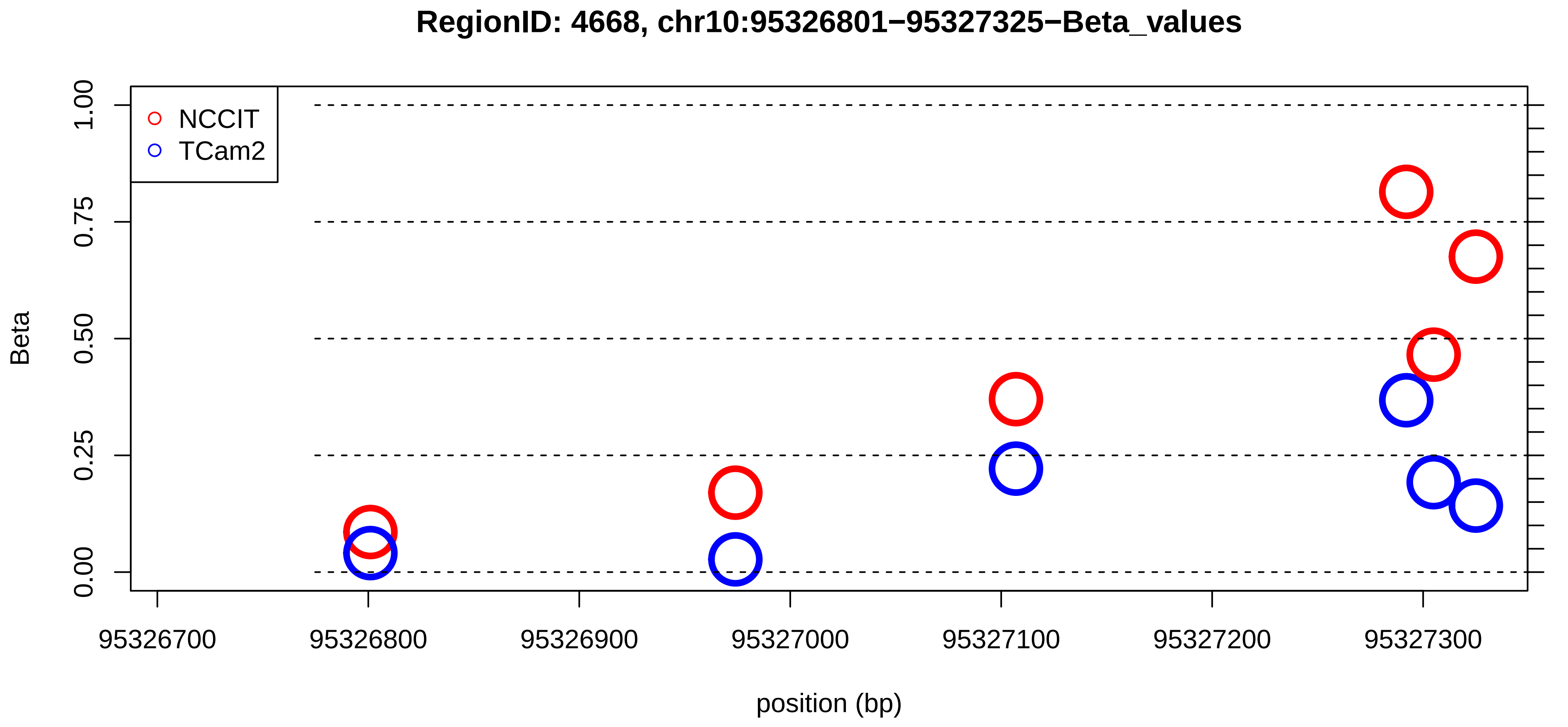
RegionID: 4668, chr10:95326801−95327325−Beta_values
NCCIT
TCam2
Beta
1.00
0.75
0.50
0.25
0.00
95326700
95326800
95326900
95327000
95327100
95327200
95327300
position (bp)
